# Clinical outcomes and genomic profiles of *MAP2K1*-mutated primary cutaneous melanocytic tumours

**DOI:** 10.1016/j.ebiom.2025.105643

**Published:** 2025-03-18

**Authors:** Chiel F. Ebbelaar, Anne M.L. Jansen, Leonie C.M. Speet, Frans Schutgens, Sietske Zoetemeyer, Anne-Marie Cleton-Jansen, Marijke R. van Dijk, Gerben E. Breimer, Lourens T. Bloem, Wendy W.J. de Leng, Remco van Doorn, Karijn P.M. Suijkerbuijk, Anne M.R. Schrader, Willeke A.M. Blokx, MOLecular Evaluation of Melanocytic Ambiguous Tumours (MOLEMAT) Investigators

**Affiliations:** a Department of Dermatology, Leiden University Medical Centre, Leiden, the Netherlands; b Department of Pathology, University Medical Centre Utrecht, Utrecht, the Netherlands; c Department of Pathology, Leiden University Medical Centre, Leiden, the Netherlands; d Department of Pathology, Symbiant, Hoorn, the Netherlands; e Department of Pathology, Reinier de Graaf Gasthuis, Delft, the Netherlands; f Division of Pharmacoepidemiology and Clinical Pharmacology, Utrecht Institute for Pharmaceutical Sciences, Utrecht University, the Netherlands; g Department of Dermatology, Netherlands Cancer Institute, Amsterdam, the Netherlands; h Department of Medical Oncology, Cancer Centre, University Medical Centre Utrecht, Utrecht, Netherlands

**Keywords:** *MAP2K1* mutations, Melanoma, Prognosis, Metastasis, Molecular oncology

## Abstract

**Background:**

Primary cutaneous melanocytic tumours harbouring *MAP2K1* mutations without second-hit genomic alterations represent a subclass of neoplasms with poorly understood biological behaviour. This study aimed to investigate the clinical outcomes and genomic characteristics of these tumours.

**Methods:**

This cohort study included primary cutaneous melanocytic tumours with *MAP2K1* mutations from patients at two academic centres (Leiden University Medical Centre and University Medical Centre Utrecht). These mutations were categorised into three functional classes: Class I (RAF-dependent), Class II (RAF-regulated), and Class III (RAF-independent). Tumours underwent histopathological evaluation, next-generation sequencing (NGS), and copy number variation (CNV) analysis and were categorised as non-melanoma or melanoma. Clinical outcomes were assessed for each mutation class during follow-up visits and through the Dutch Pathology Database (PALGA) using the composite outcome of metastatic melanoma (recurrence, metastasis, or melanoma-related death).

**Findings:**

A total of 102 patients were included, with tumours classified as melanoma in 52 (51%) and non-melanoma in 50 (49%). The tumours displayed spitzoid histomorphology in over two-thirds of cases and harboured 31 distinct *MAP2K1* mutations: 20 Class I (19.6%), 56 Class II (54.9%), and 26 Class III (25.5%). Class I mutations exclusively co-occurred with *BRAF* or *NRAS* mutations, while Class II and III mutations often acted as sole tumour drivers. Of the tumours with Class I mutations, 95% were classified as melanoma, which was less frequently the case for Class II (risk ratio [RR] 0.43 [95% CI: 0.31–0.60], p < 0.001) and Class III mutations (RR 0.40 [95% CI: 0.25–0.67], p < 0.001). *MAP2K1* mutation Class and *TERT-p* mutation status were independent predictors for the composite outcome. Compared to Class I mutations, Class II mutations were negatively associated with the composite outcome (odds ratio [OR] 0.16 [95% CI: 0.03–0.75], p = 0.03), whereas Class III mutations were not associated (OR 0.31 [95% CI: 0.05–1.54], p = 0.16). *TERT-p* mutations were positively associated with the composite outcome (OR 23.1, 95% CI: 3.99–439.8, p < 0.005).

**Interpretation:**

Class I *MAP2K1* mutations typically occur alongside other MAPK pathway mutations and may contribute to aggressive melanoma behaviour. In contrast, Class II and III *MAP2K1* mutations can independently drive melanocytic tumourigenesis with a potential for metastasis, aligning with conventional melanomagenesis pathways, despite their frequent spitzoid histomorphology.

**Funding:**

This research was supported by the 10.13039/501100023452Hanarth Fund.

**Research in context**

**Evidence before this study**

*MAP2K1* mutations have been sporadically reported across a wide range of melanocytic tumours, including common and congenital nevi, WNT-activated, protein kinase-activated, and BAP1-inactivated melanocytomas, spitzoid tumours, and (desmoplastic) melanomas. Mechanistic studies have categorised *MAP2K1* mutations into three functional classes based on their dependence on RAF phosphorylation for activation: Class I (RAF-dependent), Class II (RAF-regulated), and Class III (RAF-independent). However, the distinct prognostic implications of these mutation classes remain poorly understood.

**Added value of this study**

This study represents the largest clinical cohort to date of primary cutaneous melanocytic tumours with *MAP2K1* mutations, categorised by functional mutation classes. It provides detailed clinicopathological and molecular profiling of these tumours, highlighting the diverse clinical outcomes associated with the different classes. Class I mutations were almost exclusively found in melanomas and were always accompanied by co-driver MAPK mutations, predominantly in *BRAF* or *NRAS*, and may contribute to aggressive melanoma behaviour. In contrast, Class II and III mutations often act as sole tumourigenic drivers across the entire spectrum of melanocytic tumours, including nevi, melanocytomas, and melanomas. Class II and III driven *MAP2K1-*mutated melanomas have significant metastatic potential, particularly when accompanied by *TERT-p* mutations. These findings enhance the understanding of *MAP2K1*-mutated melanocytic tumours and contribute to refining the WHO classification of melanocytic skin tumours.

**Implications of all the available evidence**

Genomic data are increasingly incorporated in the classification of melanocytic tumours, enabling their more accurate placement within the broader context of melanomagenesis pathways. These findings have direct implications for the diagnosis, risk stratification, and management of distinct subtypes of melanocytic tumours. Future studies should focus on refining diagnostic criteria, exploring therapeutic targets specific to *MAP2K1*-driven tumours, and assessing the impact of co-occurring mutations on treatment outcomes.

## Introduction

*MAP2K1*, a proto-oncogene on chromosome 15q22 encoding Mitogen-Activated Protein Kinase (MAPK) Kinase 1 (MEK1), is a pivotal component of the RAS/RAF/MEK/ERK pathway in melanocytic tumourigenesis.^1^*MAP2K1* mutations can function as primary drivers or secondary oncogenic events and have been identified in common and congenital nevi, WNT-activated and BAP1-inactivated melanocytomas, and various types of melanomas.^2^, ^3^, ^4^, ^5^ However, cutaneous melanocytic tumours driven by *MAP2K1* mutations, without additional second hit genomic aberrations, could represent a newly identified subclass of neoplasms with a notable genotypic–phenotypic correlation not yet included in the World Health Organization (WHO) Classification of Skin Tumours.^6^ Due to their frequent spitzoid histomorphology, these neoplasms were initially proposed as a subset of Spitz tumours, alongside those driven by kinase gene fusions and *HRAS* mutations.^7^ Still, the biological behaviour of *MAP2K1*-driven melanocytic tumours remains poorly understood, and recent studies suggest they align more closely with other WHO pathways of melanomagenesis, alongside *BRAF*, *NRAS,* and *NF1* mutations.^4^^,^^8^

*MAP2K1* is a serine/threonine and tyrosine kinase that is activated by RAF kinases. Mechanistic studies have categorised *MAP2K1* mutations into three functional classes based on their dependence on phosphorylation by RAF for activation.^9^^,^^10^ Class I mutations are considered weak oncogenic drivers, likely amplifying extracellular signal-regulated kinase (ERK) signalling driven by coexisting RAS or RAF mutations. Class II mutations, which include point mutations and deletions spanning positions 51–58 (the negative regulatory region), result in mutant proteins with constitutive activity due to the loss of regulatory constraints in this region. Although these proteins are constitutively active, their activity can still be enhanced by phosphorylation through RAF. Class III mutations, involving in-frame deletions in the kinase domain (positions 98–104), produce mutant proteins with constitutive activity through autophosphorylation, rendering them entirely RAF-independent. Class III mutations are considered stronger oncogenic drivers in melanocytic tumours and are typically mutually exclusive with mutations in *BRAF, NRAS,* or *NF1*.^11^ However, the distinct prognostic implications of the different *MAP2K1* mutation classes remain unclear. In this study, we present the clinical outcomes during follow-up of patients with primary cutaneous melanocytic tumours harbouring *MAP2K1* mutations and associated genomic alterations.

## Methods

### Patients and study design

The primary objective of this multi-centre study was to assess the clinical outcomes of patients with primary cutaneous melanocytic tumours harbouring different *MAP2K1* mutation classes following initial histopathological evaluation as benign, intermediate, or malignant at baseline. The secondary objectives were to describe genomic aberrations and to assess the presence of spitzoid histomorphology and fibrosis. Patients of any age with primary cutaneous melanocytic neoplasms harbouring (likely) pathogenic *MAP2K1* mutations, identified through next-generation sequencing (NGS), were eligible for inclusion from tertiary university medical centres in Leiden (LUMC) and Utrecht (UMCU), The Netherlands. Patients with WNT-activated tumours (with deep penetrating histomorphology and additional *CTNNB1* or *APC* mutations alongside *MAP2K1* mutations) or BAP1-inactivated tumours were excluded, as these constitute established WHO categories of melanocytic neoplasms. The cohort included tumours from routine first and second line diagnostics, expert consultations submitted by peripheral pathologists in second-line settings, and revision cases referred for molecular diagnostics or second opinion.

Tumours underwent histopathological evaluation and ancillary immunohistochemistry (IHC), including melanocytic lineage markers (Melan-A, SOX-10, and S100), stainings indicative of genetic signatures (ALK, NTRK, ROS1, BAP1, and BRAFV600E), and melanoma-associated markers (HMB-45, Ki-67, p16, and PRAME), among others. Spitzoid histomorphology was defined by the presence of epithelioid or spindle-shaped melanocytes. Fibrosis, a feature associated with some *MAP2K1*-mutated melanocytic tumours, was defined as desmoplasia of the stromal tissue, characterised by dense eosinophilic collagen deposition interspersed among tumour nests.^12^ Molecular analyses, including NGS and (targeted) RNA sequencing for gene fusion analysis, were performed: i) on tumours with ambiguous histomorphology, ii) when IHC failed to identify a genetic signature, or iii) to assess the tumour’s *BRAF* status in revision melanoma cases. Single-nucleotide polymorphism (SNP) array copy number analysis was performed when feasible. Following the diagnostic workup, tumours were classified as benign (nevus), intermediate (melanoma-in-situ and melanocytoma), or malignant (melanoma).

### Molecular analyses

Amplicon-based targeted NGS was performed using Ion AmpliSeq™ custom-designed panels, including the expanded Cancer Hotspot Panels (CHP) v2plus4, CHPv4, CHPv6, or the Melanocytic Lesion Panel (MLP). All panels included amplicons covering (hotspot regions of) *APC, BRAF, CDKN2A, CTNNB1, GNA11, GNAQ, HRAS, IDH1, KRAS, MAP2K1, NRAS,* and the *TERT* promoter *(TERT-p)* region (Supplementary Table S1). *NF1* and *BAP1* were included in the CHPv6 and MLP panels.

Copy number variation (CNV) analysis was conducted using the Infinium CytoSNP-850K v1.2 BeadChip (Illumina, San Diego, CA). The CytoSNP-850K assay uses 850,000 SNP probes with enhanced coverage for 3262 cancer-relevant genes, facilitating the detection of CNVs, chromothripsis, and copy-neutral loss of heterozygosity (CN-LOH).^2^

RNA sequencing was performed with Archer FusionPlex panels, including the Comprehensive Thyroid and Lung (CTL) panel v2, the PAN Solid v2 panel, or the Melano-Lung panel. All panels included at least *ALK*, *BRAF*, *MET*, *NTRK1*, *NTRK2*, *NTRK3*, *RET*, and *ROS1*. The Melano-Lung panel also included a custom primer spike-in targeting *MAP3K3*, *MAP3K8*, *PRKCA*, and *TRIM11*.

### Classification of *MAP2K1* mutations

The identified *MAP2K1* mutations were categorised into three functional classes based on their genomic location, structural context, and published data on RAF phosphorylation dependence and intrinsic kinase activity (Supplementary Table S2).^9^, ^10^, ^11^^,^^13^ Class I mutations consist of point mutations distributed throughout the *MAP2K1* gene. These mutations result in mutant proteins that require phosphorylation by RAF to achieve activation (RAF-dependent). Class II mutations are defined by point mutations or deletions within the negative regulatory region (codons 51–58). These mutations produce mutant proteins with constitutive activity that can be further enhanced by RAF phosphorylation (RAF-regulated). Class III mutations include deletions within the kinase domain, specifically at codons 98–104. These alterations generate mutant proteins with constitutive activity driven by autophosphorylation, making them entirely independent of upstream RAF activation (RAF-independent), enhancing their oncogenic potential. Additionally, novel mutations identified within the known Class II (codons 51–58) or Class III (codons 98–104) regions were categorised into these respective classes based on their proximity to established mutational hotspots and predicted functional impact. For two *MAP2K1* mutations (p.N109_R113del and p.E102_I103delinsVN), functional data were insufficient or contradictory. Based on their location within the *MAP2K1* gene, they were included in Class III in the main analysis, with sensitivity analyses considering them as Class II.

### Follow-up and clinical outcomes

Following the diagnostic classification based upon integration of all histological, immunohistochemical and molecular findings, patients were categorised into two risk groups: the melanoma and the non-melanoma group, which included patients with benign and intermediate tumours. All patients were monitored for the occurrence of the composite outcome of metastatic melanoma, defined as local malignant recurrence, satellite or in-transit metastasis, locoregional lymph node metastasis, distant metastasis, or melanoma-related death. Outcomes were primarily documented through clinical follow-up visits when indicated, such as in patients with clinical stage II melanoma or higher. For patients with benign or intermediate tumours, or thin melanomas (Breslow thickness < 0.8 mm), for whom follow-up was not indicated, outcomes were recorded through the Dutch Nationwide Pathology Database (PALGA), which includes (molecular) pathology results, patient characteristics, autopsy results, and mortality data from all patients in the Netherlands.^14^

### Ethics

This study adhered to the institutional ethics guidelines for observational research of Leiden University Medical Centre and University Medical Centre Utrecht and complied with reporting recommendations. Under Dutch regulations in the Medical Research Involving Human Subjects Act, written informed consent was not required as no procedures beyond standard diagnostics were performed, and the institutional review board formally waived this requirement. An opt-out system was used, and one patient withdrew and was excluded from the analysis.

### Statistical analyses

We aimed to include a minimum of 100 patients with *MAP2K1*-mutated tumours to ensure a sufficiently representative group for analysing the distribution of *MAP2K1* mutation classes and their associated histopathological and clinical features. Therefore, a formal sample size calculation was not performed. Data were analysed descriptively. In addition, multiple logistic regression analyses were performed to assess whether *MAP2K1* mutation class and additional oncogenic alterations including *TERT-p* mutations, *TP53* mutations and *IDH1* mutations were predictors for the composite outcome comprising malignant recurrence, metastatic melanoma, and melanoma-related death by estimating odds ratios (OR) and 95% confidence intervals (CIs). The full model (i.e., including all four covariates) was reduced using the likelihood ratio test to identify the best fitting model (i.e., including the covariates with predictive capacity). Also, chi-square tests were performed to assess potential associations between i) *MAP2K1* mutation class and tumour classification (melanoma vs. non-melanoma); ii) fibrosis and *MAP2K1* mutation class; and iii) spitzoid histomorphology and *MAP2K1* mutation class. If the results of the chi-square test indicated an association, risk ratios (RR), 95% Cis, and Fisher’s exact p values were estimated.

### Role of funders

The funding sources were not involved in the study design, data collection, data analysis, interpretation of results, or the writing of the report.

## Results

Between 1 October 2019 and 1 October 2024 NGS was performed on a total of 2288 primary cutaneous melanocytic tumours. Of these, *MAP2K1* mutations, without concurrent WNT activation or *BAP1* inactivation, were identified in 103 patients (4.5%). The cohort comprised 49.0% consultation cases submitted by peripheral pathologists in second-line settings, 39.2% routine diagnostic cases (including first and second line), and 11.8% revision cases referred for molecular diagnostics or second opinion. One patient withdrew consent, resulting in 102 patients available for analysis. The disease course of four of these patients has been previously published.^7^ Baseline characteristics, management, and clinical outcomes of all patients are shown in Table 1. The median age was 52 years (interquartile range [IQR] 36–62 years), with 53.9% of patients being female, and 57.8% of the tumours localised to the extremities. The median follow-up for all patients was 28 months (IQR 12–48 months; Table 1). Following histopathological evaluation and molecular analyses, 28 tumours were classified as benign (27.5%), 22 as intermediate (21.6%), and 52 as malignant (51.0%). Among the 50 patients in the non-melanoma group, two had *MAP2K1*-driven tumours originating from a medium-sized congenital nevus and a nevus spilus. In the melanoma group, most tumours were superficial spreading melanomas (n = 43; 82.7%), followed by nodular melanomas (n = 7; 13.5%) and lentigo maligna melanomas (n = 2; 3.8%).

**Table 1:** Baseline characteristics, management, and clinical outcomes of patients with primary cutaneous melanocytic tumours with *MAP2K1* mutations.

*MAP2K1* mutation	Non-melanoma group (n = 50)			Melanoma group (n = 52)			Total n = 102
	Class I n = 1	Class II n = 33	Class III n = 16	Class I n = 19	Class II n = 23	Class III n = 10	
**Sex–no. (%)**^a^							
Male	0 (0.0)	13 (39.4)	4 (25.0)	10 (52.6)	15 (65.2)	5 (50.0)	47 (46.1)
Female	1 (100.0)	20 (60.6)	12 (75.0)	9 (47.4)	8 (34.8)	5 (50.0)	55 (53.9)
**Median age–yr (IQR)**	36 (36–36)	42 (30–56)	41 (17–72)	55 (46–70)	52 (45–69)	66 (56–70)	52 (36–62)
**Localization–no. (%)**							
Extremity	0 (0.0)	27 (81.8)	10 (62.5)	4 (21.1)	13 (56.5)	5 (50.0)	59 (57.8)
Head/neck	1 (100.0)	0 (0.0)	0 (0.0)	10 (52.6)	2 (8.7)	1 (10.0)	14 (13.7)
Trunk	0 (0.0)	6 (18.2)	6 (37.5)	5 (26.3)	8 (34.8)	4 (40.0)	29 (28.4)
**Tumour**							
Tumour diameter ≥ 6 mm	1 (100.0)	17 (51.5)	7 (43.8)	13 (68.4)	14 (60.9)	7 (70.0)	59 (57.8)
Spitzoid histomorphology	0 (0.0)	28 (84.8)	12 (75.0)	10 (52.6)	16 (69.6)	5 (50.0)	71 (69.6)
Fibrosis	1 (100.0)	11 (33.3)	5 (31.3)	1 (5.3)	6 (26.1)	3 (30.0)	27 (26.5)
**Pathological staging**							
pT1a	N/A	N/A	N/A	4 (21.1)	12 (52.2)	4 (40.0)	20 (38.5)^b^
pT1b, pT2a, pT2b	N/A	N/A	N/A	3 (15.8)	9 (39.1)	3 (30.0)	15 (28.9)^b^
pT3a, pT3b	N/A	N/A	N/A	5 (26.3)	1 (4.3)	2 (20.0)	8 (15.4)^b^
pT4a, pT4b	N/A	N/A	N/A	7 (36.8)	1 (4.3)	1 (10.0)	9 (17.3)^b^
**Median follow-up–mo (IQR)**	18 (18–18)	25 (6–41)	31 (8–57)	30 (16–40)	39 (18–46)	28 (10–55)	28 (12–42)
**Re-excision–no. (%)**	0 (0.0)	8 (23.5)	7 (46.7)	18 (94.7)	24 (100.0)	9 (100.0)	47 (65.3)
**Positive SNB–no./total no. (%)**	0/0 (0.0)	0/0 (0.0)	0/0 (0.0)	6/10 (60.0)	0/8 (0.0)	1/4 (25.0)	7/22 (31.8)
**Clinical staging**							
IA	N/A	N/A	N/A	4 (21.1)	12 (52.2)	4 (40.0)	20 (38.5)^b^
IB–IIC	N/A	N/A	N/A	7 (36.8)	8 (34.8)	3 (30.0)	18 (34.6)^b^
IIIA–IIIC	N/A	N/A	N/A	5 (26.3)	1 (4.3)	0 (0.0)	6 (11.5)^b^
IV	N/A	N/A	N/A	3 (15.8)	2 (8.7)	3 (30.0)	8 (15.4)^b^
**Metastatic melanoma–no. (%)**	0 (0.0)	0 (0.0)	0 (0.0)	9 (47.4)	3 (9.1)	3 (30.0)	15 (14.7)
Recurrence	0 (0.0)	0 (0.0)	0 (0.0)	1 (5.3)	0 (0.0)	0 (0.0)	1 (1.0)
Satellite metastasis	0 (0.0)	0 (0.0)	0 (0.0)	1 (5.3)	0 (0.0)	0 (0.0)	1 (1.0)
In-transit metastasis	0 (0.0)	0 (0.0)	0 (0.0)	0 (0.0)	1 (4.3)	0 (0.0)	1 (1.0)
Lymph node metastasis	0 (0.0)	0 (0.0)	0 (0.0)	4 (21.1)	0 (0.0)	0 (0.0)	4 (3.9)
Distant metastasis	0 (0.0)	0 (0.0)	0 (0.0)	0 (0.0)	2 (8.7)	1 (10.0)	4 (3.9)
Melanoma-related death	0 (0.0)	0 (0.0)	0 (0.0)	3 (15.8)	0 (0.0)	2 (20.0)	4 (3.9)
**Other mortality**	0 (0.0)	0 (0.0)	1 (6.7)	0 (0.0)	1 (4.3)	0 (0.0)	2 (2.0)

IQR: interquartile range. SNB: Sentinel Node Biopsy.

a Sex was self-reported.

b Denominator based on the melanoma group (n = 52).

### *MAP2K1* mutations

For all cases, sufficient tumour tissue was available for next-generation sequencing. A total of 31 distinct *MAP2K1* mutations were identified (Supplementary Table S2), with 20 tumours (19.6%) harbouring Class I mutations, 56 tumours (54.9%) having Class II mutations, and 26 tumours (25.5%) having Class III mutations (Table 1). These included four distinct Class I mutations, primarily p.P124S (n = 10; 50.0%) and p.P124L (n = 7; 35.0%); 24 distinct Class II mutations, predominantly p.E203K (n = 9; 16.1%) and p.Q58_E62del (n = 9; 16.1%), and three different Class III mutations, primarily p.I103_K104del (n = 14; 53.8%) and p.E102_I103del (n = 9; 34.6%).

Of the tumours with Class I mutations, 95% (19/20) were classified as melanoma, which was less frequently the case for Class II (23/56; 41%, RR 0.43 [95% CI: 0.31–0.60], Fisher-exact p < 0.001) and Class III mutations (10/26; 38%, RR 0.40 [95% CI: 0.25–0.67], Fisher-exact p < 0.001). The sensitivity analyses, including p.N109_R113del and p.E102_I103delinsVN as Class II mutations, did not substantially change the results. In 74 tumours (72.5%), *MAP2K1* mutations, all classified as either Class II or III, were the sole identified drivers.

**Fig. 1:**
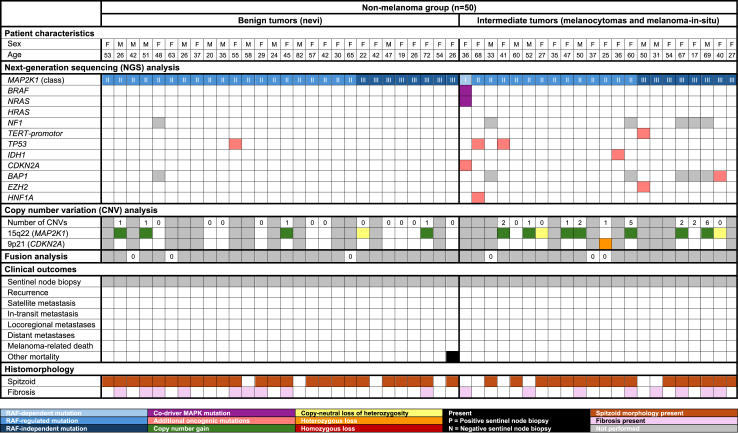
Patient characteristics, genomic findings, clinical outcomes and histomorphology of patients in the non-melanoma group.

**Fig. 2:**
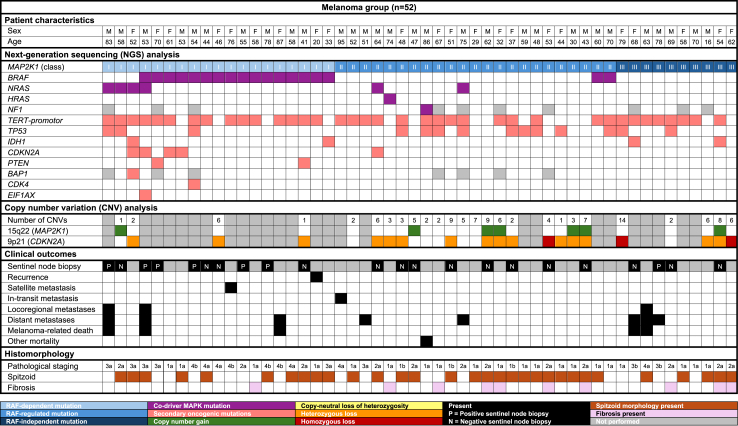
Patient characteristics, genomic findings, clinical outcomes and histomorphology of patients in the melanoma group.

All tumours with Class I mutations exhibited concomitant *BRAF* or *NRAS* mutations (Figs. 1 and 2). With the exception of one melanocytoma, these tumours were classified as melanoma. Notably, two patients with phenotypically heterogeneous tumours harbouring *MAP2K1*^*D67N*^ mutations had additional *BRAF* and *NRAS* mutations: one with a melanocytoma containing *BRAF*^*V600E*^ and *NRAS*^*Q61R*^, and another with a melanoma containing *BRAF*^*G469R*^ and *NRAS*^*Q61R*^ mutations. Of the 56 tumours with Class II mutations, six (10.7%), all classified as melanoma, had additional MAPK pathway mutations, including *BRAF*^*K601E*^, *BRAF*^*G469A*^, *NRAS*^*Q61K*^, *NRAS*^*G12D*^, *HRAS*^*Q61K*^, and *NF1*^*W1559*^*∗* (NF1 was not included in the NGS panel for 16 tumours).

None of the tumours with Class III *MAP2K1* mutations had co-driver mutations.

### Spitzoid histomorphology and fibrosis

In total, 71 tumours (69.6%) exhibited spitzoid histomorphology (Table 1 and Supplementary Figs. S1–S3), with no difference between *MAP2K1* mutation classes (*X*^2^ (2, *N* = 102) = 6.0, p = 0.05). Among the 76 tumours driven solely by Class II or III *MAP2K1* mutations, 59 (77.6%) showed spitzoid histomorphology. In contrast, only 10 out of 20 tumours (50.0%) with Class I *MAP2K1* mutations and additional MAPK pathway mutations exhibited spitzoid histomorphology. A total of 27 tumours (26.5%) showed stromal fibrosis (Table 1 and Supplementary Fig. S1),^12^ There was no association between *MAP2K1* mutation class and the presence of fibrosis (*X*^2^ (2, *N* = 102) = 3.5, p = 0.18).

### Genomic alterations

The most common additional pathogenic mutations were *TERT-p* (n = 38; 37.3%), *TP53* (n = 17; 16.7%), and *IDH1*^*R132C*^ (n = 5; 4.9%), see Figs. 1 and 2. With the exception of one melanocytoma, all tumours with *TERT-p* mutations were classified as melanoma. Six tumours (5.9%) had a *CDKN2A* mutation, three of which were in patients with the germline p16-Leiden mutation. RNA sequencing, performed in six tumours with spitzoid morphology, did not identify additional kinase gene fusions.

Copy number analysis was successfully performed in 53 (51.9%) tumours (Figs. 1 and 2 and Supplementary Table S3). The median number of CNVs was 0 (IQR 0–1) in benign tumours, 2 (IQR 1–2) in intermediate tumours, and 4.5 (IQR 2–6) in malignant tumours. SNP array identified copy number alterations in 15q22 (*MAP2K1*) in 21 tumours (38.9%), with 14 (66.7%) exhibiting stromal fibrosis, compared to only 7 of 33 tumours (21.2%) without gain of 15q22. In 6 tumours, all classified as nevus or melanocytoma, gain of 15q22 was the sole copy number aberration (Fig. 1). Additionally, 19 tumours (35.2%) exhibited loss of 9p21 (*CDKN2A*), including three with homozygous deletion, and all but one were classified as melanoma (Fig. 2). Other frequent copy number aberrations included gain of 6p (n = 5) and loss of 6q (n = 5), all occurring in melanocytomas or melanomas (Supplementary Table S3).

### Clinical outcomes

Of the 50 patients in the non-melanoma group, 15 (30.0%) underwent re-excision (Table 1). None of these patients developed lymph node or distant metastases during a median follow-up of 27 months (IQR 8–42 months), see Fig. 1. One patient died of causes unrelated to melanoma.

In the melanoma group (n = 52), all but one patient underwent re-excision (Table 1). Among the 32 patients with pT1b-pT4b melanomas, 22 (68.8%) had sentinel lymph node procedures, with positive results in 7 (31.8%), see Fig. 2. Fine needle aspirations were performed in 11 patients, yielding positive results in 4 (36.4%). During a median follow-up of 32 months (IQR 15–46 months), 15 melanoma patients (28.8%), all with pT2a–pT4b tumours, developed metastatic melanoma: nine with Class I, three with Class II, and three with Class III *MAP2K1* mutations (Table 1 and Fig. 2). The final logistic regression model indicated that *MAP2K1* mutation Class and *TERT-p* mutation status were independent predictors for the composite outcome (Supplementary Table S4). Compared to Class I mutations, Class II mutations were negatively associated with the composite outcome (OR 0.16, 95% CI: 0.03–0.75, p = 0.03), whereas Class III mutations were not associated (OR 0.31, 95% CI: 0.05–1.54, p = 0.16). All six patients who developed distant metastases had tumours harbouring additional *TERT-p* mutations (Fig. 2). *TERT-p* mutations were positively associated with the composite outcome (OR 23.1, 95% CI: 3.99–439.8, p < 0.005). This association was not significant for *TP53* or *IDH1* mutations. The sensitivity analyses, including p.N109_R113del and p.E102_I103delinsVN as class II mutations, did not substantially change the results. One patient died from causes unrelated to melanoma.

Of the 19 melanoma patients with Class I mutations, three (15.8%) developed distant metastases and subsequently died from melanoma (Table 1). In one patient who died of melanoma, systemic treatment was withheld due to frailty. The second patient, with an additional *NRAS*^*G13C*^ mutation, died following nivolumab failure. The third patient died after developing brain metastases. One patient with stage IIIC melanoma harbouring *BRAF*^*V600K*^ and *MAP2K1*^*P124L*^ mutations achieved persistent metabolic remission after adjuvant dabrafenib/trametinib treatment.

Among the 23 melanoma patients with Class II mutations, three (13.0%) developed metastatic melanoma: one with a *MAP2K1*^*E203K*^ mutation developed cerebral metastases, another with *MAP2K1*^*G128D*^ and *NRAS*^*G12D*^ mutations developed bone metastases, and a third with a *MAP2K1*^*C121S*^ mutation had inoperable in-transit metastases treated with palliative radiotherapy. None of these patients died during follow-up.

Among the 10 melanoma patients with Class III mutations, three (30.0%) with *MAP2K1*^*E102_I103del*^ mutations developed bone, lung, and liver metastases, with two subsequently dying after pembrolizumab and ipilimumab treatment failure.

## Discussion

This study highlights the clinicopathological and molecular features of the largest patient cohort with primary cutaneous melanocytic tumours harbouring different classes of *MAP2K1* mutations, with a median follow-up of over two years. Our findings demonstrate the diversity of these tumours, ranging from (congenital) nevi and nevus spilus to melanocytomas, melanoma-in-situ, and melanomas. Class I *MAP2K1* mutations, which were consistently accompanied by co-driver MAPK pathway mutations, were almost exclusively found in melanomas and were strongly associated with metastatic disease. In contrast, Class II and III mutations predominantly acted as sole drivers. Despite the absence of additional MAPK mutations, melanomas with Class II and III *MAP2K1* mutations progressed to metastasis in one-eighth and one-third of cases, respectively, with the presence of *TERT-p* mutations being an independent predictor for metastatic disease. These results show that Class II and III *MAP2K1* mutations can initiate melanocytic tumourigenesis with additional oncogenic alterations, particularly *TERT-p* mutations and loss of 9p21 (*CDKN2A*), driving malignant progression.

The clinical outcomes in our study align with mechanistic research that categorises *MAP2K1* mutations into three functional classes based on their dependence on RAF-mediated phosphorylation for activation.^9^^,^^10^ Class I mutations, with p.P124L and p.P124S being the most common in our study, are fully RAF-dependent and likely act as amplifiers of ERK signalling when coexisting with other mutations. None of the tumours in our study had Class I mutations as their sole primary driver, suggesting these mutations alone are insufficient for melanocytic tumourigenesis. This is consistent with previous studies on benign and intermediate melanocytic tumours with *MAP2K1* mutations, where a tumour with a Class I mutation also had a co-driver *HRAS* mutation.^4^ In melanomas, Class I *MAP2K1* mutations have been identified as secondary oncogenic drivers, typically occurring alongside *BRAF*, *NRAS*, or *NF1* mutations.^1^ This aligns with our findings, where all tumours with Class I mutations harboured co-driving *BRAF* or *NRAS* mutations. Almost half of the melanoma patients with Class I mutations developed locoregional or distant metastases or died. Although Class I mutations may not serve as primary drivers, they might play a role in tumour progression and poor clinical outcomes when combined with other oncogenic alterations.

Beyond melanoma, Class II and III *MAP2K1* mutations have been identified in spitzoid, WNT-activated, protein kinase-activated, and BAP1-inactivated tumours, but reports of *MAP2K1*-driven melanocytic neoplasms without additional molecular alterations are scarce, with limited clinical follow-up.^4^^,^^15^, ^16^, ^17^ Our study shows that both Class II and III *MAP2K1* mutations can serve as primary drivers in nevi, melanocytomas, and melanomas in the absence of co-drivers in the MAPK pathway. Class II mutations (RAF-regulated) include point mutations and deletions in the negative regulatory 51–58 region, while Class III mutations are RAF-independent, with constitutive kinase activity and consist mainly of in-frame deletions in the 98–104 kinase domain. The latter have been proposed as a unique subset of triple wild-type melanoma, aligning with our findings that these mutations are mutually exclusive with *RAS*, *RAF*, and *NF1* mutations.^11^ Our study demonstrates that Class II and Class III *MAP2K1* mutations have the potential to initiate aggressive tumour behaviour without concurrent MAPK mutations, as evidenced by patients who developed widespread distant metastases and subsequently died. Aggressive behaviour of Class II or III *MAP2K1*-driven tumours is likely stimulated by additional oncogenic alterations, as all these melanomas also harboured *TERT-p* mutations.

In our study, Class II and III *MAP2K1*-driven tumours exhibited spitzoid histomorphology in all nevi and three-quarters of melanocytomas and melanomas, compared to half of the tumours with Class I *MAP2K1* mutations and concomitant MAPK mutations. An important question is whether *MAP2K1*-driven tumours align with conventional WHO melanomagenesis pathways, alongside *BRAF, NRAS*, and *NF1* mutations, or pathway IV (Spitz), alongside kinase gene fusions and *HRAS* mutations. The median ages of patients with Class II and III *MAP2K1* mutations, in both the non-melanoma and melanoma groups, were notably higher than in the typically younger individuals with Spitz tumours. Also, Spitz melanomas are thought to generally have a more favourable prognosis than melanomas with *BRAF*, *NRAS*, or *NF1* mutations.^18^ Our clinical and genomic findings suggest that Class II and III *MAP2K1*-driven tumours align more closely with conventional WHO pathways of melanomagenesis.

A key strength of this study is that it represents the largest clinical cohort of patients with genetically characterised *MAP2K1*-driven primary cutaneous melanocytic tumours to date, coupled with detailed follow-up data. However, several limitations should be noted. First, although we aimed to comprehensively characterise the tumours for mutations, copy number variations, and gene fusions using NGS, SNP-array, and RNA sequencing, we cannot entirely rule out the possibility that clinically relevant molecular alterations were overlooked. For instance, *NF1* was not included in the initial NGS panels, which may have led to missed co-occurring MAPK mutations in a minority of tumours. Additionally, CNV analysis was performed in slightly more than half of the cases, potentially impacting tumour classification. Fusion analysis was performed in only a minority of tumours, leaving the possibility that co-driver kinase fusions may have been missed. However, kinase fusions and MAPK mutations rarely co-occur. Second, the relatively short follow-up duration may have resulted in an underestimation of late-occurring metastatic melanoma. Nonetheless, previous studies in stage I to III melanoma patients have demonstrated that the peak incidence of metastases occurs early, with approximately 80% identified within the first three years after diagnosis.^19^ Third, the documentation of metastatic melanoma outcomes relied partly on registry data rather than prospectively planned hospital visits. However, the PALGA database is a comprehensive, nationwide resource that includes all pathology reports and mortality data in the Netherlands, providing a robust database for research. Given its thorough coverage, it is unlikely that we missed significant clinical events. Fourth, molecular analyses were performed based on clinicopathological indications rather than uniformly across all tumours, which may have led to an overrepresentation of clinically unfavourable or diagnostically atypical tumours. This could introduce selection bias and potentially overestimate poor prognostic findings, thereby limiting the generalisability of the results. However, the cohort includes a broad range of clinical scenarios, predominantly routine first- and second-line diagnostic cases, as well as expert consultations from peripheral pathologists. A smaller proportion comprised revision cases referred specifically for molecular diagnostics. Thus, we believe the cohort is sufficiently representative of clinical practice. Finally, it should be acknowledged that a relatively small sample size was analysed in this study. While this study represents the largest cohort of *MAP2K1*-driven primary cutaneous melanocytic tumours to date, larger studies in the future will be essential to validate and strengthen these findings.

In conclusion, this study highlights the diverse clinical and genomic features of primary cutaneous melanocytic tumours with *MAP2K1* mutations, emphasizing the role of specific mutation classes and additional pathogenic alterations in driving aggressive behaviour and metastasis. Class I *MAP2K1* mutations typically occur alongside other MAPK pathway mutations and may contribute to aggressive melanoma behaviour. In contrast, Class II and III *MAP2K1* mutations can independently drive melanocytic tumourigenesis with a potential for metastasis, aligning with conventional melanomagenesis pathways, despite their frequent spitzoid histomorphology.

## Contributors

Conceptualisation: CE, AJ, AS, WB; methodology: CE, LB, AJ, AS, WB. Data curation: CE, AJ, LS, FS, SS, AC, MD, GB, WL, RD, KS, AS, WB; verification of the underlying data: CE, LS, AJ, WB; formal analysis: CE; LB; resources: SF, LF, TE, CG, FM, IB, DLR, MR; visualisation: CE; supervision: AJ, AS, WB; funding acquisition: WB; writing—original draft: CE; writing—review & editing: all authors. All authors read and approved the final version of the manuscript.

## Data sharing statement

All data are available from the corresponding author upon reasonable request after an appropriate data access agreement specifying the terms and conditions of use of the data.

## Declaration of interests

KS has received grants or contracts from Philips, Bristol Myers Squibb, Genmab, TigaTx, and Pierre Fabre, received consulting fees from Abbvie and support for attending meetings and/or travel from Bristol Myers Squibb, and has participated on a Data Safety Monitoring Board or Advisory Board for Sairopa. All other authors have no conflicts of interest to declare. The MOLEMAT consortium has not received any additional funding beyond what is already disclosed in the manuscript.
