# Supplementary material for: Clinical outcomes and genomic profiles of MAP2K1-mutated primary cutaneous melanocytic tumours
Source: eBioMedicine. 2025 Mar 18;114:105643. doi: 10.1016/j.ebiom.2025.105643 (PMC11960658; doi:10.1016/j.ebiom.2025.105643)
Supplement: Supplementary Figs. S1–S3 and Tables S1–S4 [file mmc1.docx]

**Table S1. Next-generation sequencing (NGS) panels.**

| **NGS panel** | **Included (hotspots of) genes** |
| --- | --- |
| Cancer Hotspot Panels **(**CHP) v2plus4 | *ABL1, AKT1, ALK, AMELY, APC, ARAF, ATM, BRAF, CALR, CCND1, CDH1, CDK4, CDKN2A, CSF1R, CTNNB1, DDX3Y, EGFR, ERBB2, ERBB4, EZH2, FBXW7, FGFR1, FGFR2, FGFR3, FLT3, GNA11, GNAQ, GNAS, HNF1A, HRAS, IDH1, IDH2, JAK2, JAK3, KDR, KIT, KRAS, MAP2K1, MDM2, MET, MLH1, MPL, MYD88, NOTCH1, NPM1, NRAS, PDGFRA, PIK3CA, POLD1, POLE, PTEN, PTPN11, RAF1, RB1, RET, ROS1, SMAD4, SMARCB1, SMO, SRC, STK11, TERT, TP53, VHL* |
| Cancer Hotspot Panels (CHP) v4 | *ARAF, CD79B, CIC, CTNNB1, EIF1AX, ERBB3, KRAS, NRAS, HRAS, BRAF, EGFR, GNAQ, GNAS, H3F3A, H3F3B, IDH1, IDH2, KIT, MAP2K1, MAP2K2, MAP2K4, MAP3K1, MDM2, MED12, MYD88, MUTYH, PDGFRA, PDGFRB, PIK3CA, POLE, RET, TP53, ABL1, AKT1, ALK, APC, ATM, CARD11, CD79A, CDK4, CDH1, CDKN2A, CSF1R, CTNNB1, ERBB2, ERBB4, EZH2, FBXW7, FGFR1, FGFR2, FGFR3, FLT3, FOXL2, GNA11, HNF1A, JAK2, JAK3, KDR, MET, MLH1, MPL, MYC, NOTCH1, NPM1, PTEN, PTK2, PTPN11, RB1, SMAD4, SMARCB1, SMO, SRC, STK11, TERT, VHL.* |
| Cancer Hotspot Panels (CHP) v6 | *ABL1, AKT1, ALK, APC, ARAF, ATM, BAP1, BRAF, CARD11, CD79A, CD79B, CDC73, CDH1, CDK4, CDKN2A, CIC, CSF1R, CTNNB1, DDR, DICER, EGFR, EIF1AX, ERBB2, ERBB3, ERBB4, ERCC2, EZH2, FBXW7, FGFR1, FGFR2, FGFR3, FLT3, FOXL2, GNA11, GNAQ, GNAS, H3F3A, H3F3B, HNF1A, HRAS, IDH1, IDH2, JAK2, JAK3, KDR, KIT, KRAS, MAP2K1, MAP2K2, MAP2K4, MAP3K1, MDM2, MED12, MET, MLH1, MPL, MUTYH, MYC, MYD88, MyoD1, NF1, NKX2-1, NOTCH1, NTRK1, NPM1, NRAS, PDGFRA, PDGFRB, PIK3CA, POLD1, POLE, PPP2R1A, PTEN, PTK2, PTPN11, RB1, RET, SMAD4, SMARCB1, SMO, SRC, STK11, TERT, TP53, VHL.* |
| Melanocytic Lesion Panel (MLP) | *APC, BAP1, BRAF, CCND1, CDKN2A, CTNNB1, CYSLTR2, EGFR, EIF1AX, ERBB2, GNA11, GNAQ, GNAS, HRAS, IDH1, IDH2, JAK1, JAK2, KIT, KRAS, MAP2K1, MAP2K2, MET, NF1, NRAS, PLCB4, PRKAR1A, PTEN, SF3B1, TERT, TP53* |

**Table S2. Identified *MAP2K1* mutations and their functional classification.**

| ***MAP2K1* mutation** | **Mutation type** | **Class** | **Reason for classification**^1–4^ | **N (%)** |
| --- | --- | --- | --- | --- |
| F53C | Missense | II | Mutation in negative regulatory domain (51-58) | 1 (1.0) |
| F53L | Missense | II | Mutation in negative regulatory domain (51-58) | 2 (2.0) |
| F53_Q58delinsL | In-frame deletion and insertion | II | Deletion-insertion in negative regulatory domain (51-58) | 2 (2.0) |
| Q56P | Missense mutation | II | Mutation in negative regulatory domain (51-58) | 1 (1.0) |
| Q56_V60del | In-frame deletion | II | Deletion involving the negative regulatory domain (51-58) | 3 (2.9) |
| Q56_G61delinsP | In-frame deletion and insertion | II | Deletion-insertion involving the negative regulatory domain (51-58) | 5 (4.9) |
| Q56_G61delinsR | In-frame deletion and insertion | II | Deletion-insertion involving the negative regulatory domain (51-58) | 1 (1.0) |
| K57E | Missense mutation | II | Previously described and determined with functional assays | 1 (1.0) |
| K57N | Missense mutation | II | Previously described and determined with functional assays | 1 (1.0) |
| K57_Q58delinsNS | Double missense mutation | II | Expected similar activation as K57N | 1 (1.0) |
| K57_G61del | In-frame deletion | II | Deletion involving the negative regulatory domain (51-58) | 1 (1.0) |
| K57_E62delinsN | In-frame deletion and insertion | II | Deletion-insertion involving the negative regulatory domain (51-58) | 1 (1.0) |
| Q58_E62del | In-frame deletion | II | Previously described and determined with functional assays | 9 (8.8) |
| D67N | Missense mutation | I | Previously described and determined with functional assays | 2 (2.0) |
| I99_I103del | In-frame deletion | III | Deletion in the 98-104 region | 1 (1.0) |
| E102_I103del | In-frame deletion | III | Deletion in the 98-104 region | 9 (8.8) |
| E102_I103delinsVN | Double missense mutation | III | Functional data were contradictory. Expected classification based on location | 1 (1.0) |
| I103_K104del | In-frame deletion | III | Deletion in the 98-104 region | 14 (13.7) |
| K104_A106delinsT | In-frame deletion and insertion | II | Expected classification based on location and findings related to p.P105_A106del | 4 (3.9) |
| N109_R113del | In-frame deletion | III | Functional data were contradictory. Expected classification based on location | 1 (1.0) |
| P105_A106del | In-frame deletion | II | Previously described and determined with functional assays | 8 (7.8) |
| P105_I107delinsL | In-frame deletion and insertion | II | Expected classification based on location and findings related to p.P105_A106del | 1 (1.0) |
| P105_I107delinsN | In-frame deletion and insertion | II | Expected classification based on location and findings related to p.P105_A106del | 1 (1.0) |
| P105_I107delinsS | In-frame deletion and insertion | II | Expected classification based on location and findings related to p.P105_A106del | 1 (1.0) |
| C121S | Missense mutation | II | Previously described and determined with functional assays | 1 (1.0) |
| P124L | Missense mutation | I | Previously described and determined with functional assays | 7 (6.9) |
| P124S | Missense mutation | I | Previously described and determined with functional assays | 10 (9.8) |
| G128D | Missense mutation | II | Previously described and determined with functional assays | 1 (1.0) |
| Y130C | Missense mutation | I | Previously described and determined with functional assays | 1 (1.0) |
| E203K | Missense mutation | II | Previously described and determined with functional assays | 9 (8.8) |
| E203_I204delinsKT | Double missense mutation | II | Expected classification based on location and findings related to E203K | 1 (1.0) |
| **Total** |  |  |  | **102** |

1. Mizuno, S. et al. High-Throughput Functional Evaluation of MAP2K1 Variants in Cancer. Mol Cancer Ther 22, 227 (2023).

2. Gao, Y. et al. Allele-Specific Mechanisms of Activation of MEK1 Mutants Determine Their Properties. Cancer Discov 8, 648–661(2018).

3. Williams, E. A. et al. Melanoma with in-frame deletion of MAP2K1: a distinct molecular subtype of cutaneous melanoma mutually exclusive from BRAF, NRAS, and NF1 mutations. Mod Pathol 33, 2397–2406 (2020).

4. Hanrahan, A. J. et al. Leveraging Systematic Functional Analysis to Benchmark an In Silico Framework Distinguishes Driver from Passenger MEK Mutants in Cancer. Cancer Res 80, 4233–4243 (2020).

**Table S3. Identified copy number variations for all patients.**

| **ID** | **Method** | **Copy number variations (CNVs)** |
| --- | --- | --- |
| B01 | Not performed | - |
| B02 | SNP array | +partial 15q |
| B03 | Not performed | - |
| B04 | SNP array | Trisomy 15 |
| B05 | SNP array | No abnormalities detected |
| B06 | Not performed | - |
| B07 | Not performed | - |
| B08 | Not performed | - |
| B09 | SNP array | No abnormalities detected |
| B10 | SNP array | No abnormalities detected |
| B11 | Not performed | - |
| B12 | Not performed | - |
| B13 | SNP array | Partial CN-LOH 22q |
| B14 | Not performed | - |
| B15 | SNP array | Trisomy 15 |
| B16 | Not performed | - |
| B17 | SNP array | No abnormalities detected |
| B18 | SNP array | No abnormalities detected |
| B19 | Not performed | - |
| B20 | Not performed | - |
| B21 | SNP array | CN-LOH 15q |
| B22 | Not performed | - |
| B23 | SNP array | No abnormalities detected |
| B24 | SNP array | No abnormalities detected |
| B25 | SNP array | +partial11q |
| B26 | SNP array | Trisomy 15 |
| B27 | Not performed | - |
| B28 | SNP array | No abnormalities detected |
| I01 | Not performed | - |
| I02 | Not performed | - |
| I03 | Not performed | - |
| I04 | SNP array | +6p, +partial 15q |
| I05 | SNP array | No abnormalities detected |
| I06 | SNP array | Trisomy 15 |
| I07 | SNP array | CN-LOH of chromosome 15q |
| I08 | Not performed | - |
| I09 | SNP array | Trisomy 15 |
| I10 | SNP array | -partial 3p (including 3p21), trisomy 15 |
| I11 | Not performed | - |
| I12 | SNP array | Monosomy 9 (including heterozygous loss of 9p21) |
| I13 | Not performed | - |
| I14 | SNP array | -partial 6q, trisomy 15, trisomy 20, trisomy 21, monosomy X |
| I15 | Not performed | - |
| I16 | Not performed | - |
| I17 | Not performed | - |
| I18 | SNP array | Partial AI 7q, trisomy 15 |
| I19 | SNP array | -partial 2p, -partial 19p, partial CN-LOH 19p, chromothripsis 22q |
| I20 | SNP array | Trisomy of chromosomes 5, 6, 11, 14, 15, 20 |
| I21 | SNP array | partiële CN-LOH of chromosome 15q |
| I22 | Not performed | - |
| M01 | Not performed | - |
| M02 | SNP array | partial AI 15q |
| M03 | SNP array | Monosomy 9 (including heterozygous loss of 9p21), -partial 11q |
| M04 | Not performed | - |
| M05 | Not performed | - |
| M06 | Not performed | - |
| M07 | Not performed | - |
| M08 | Not performed | - |
| M09 | Not performed | - |
| M10 | SNP array | -3p, -9p21, -1p, -5p, -10q, -partial12p |
| M11 | Not performed | - |
| M12 | Not performed | - |
| M13 | Not performed | - |
| M14 | Not performed | - |
| M15 | Not performed | - |
| M16 | Not performed | - |
| M17 | SNP array | -partial 9p (including heterozygous loss of 9p21) |
| M18 | Not performed | - |
| M19 | Not performed | - |
| M20 | Not performed | - |
| M21 | SNP array | -partial 14q, +partial 17q |
| M22 | Not performed | - |
| M23 | SNP array | -9p (including heterozygous loss of 9p21), partial AI 2q, -17p, -20p, - partial 20q, +partial 20q |
| M24 | SNP array | Monosomy 9, -partial 1q, monosomy X |
| M25 | SNP array | Monosomy 9, AI 11p, AI 17p |
| M26 | SNP array | -partial 6q, +7p, +partial 15q (including 15q22), -17p, +17q |
| M27 | SNP array | Monosomy 7, partial AI 11q |
| M28 | SNP array | -partial 5p, -partial 12q |
| M29 | SNP array | -9p (including 9p21), partial CN-LOH 1p, +partial 2q, +partial 4q, +partial 20q, -partial 6q, -9q, -13q, -14q, monosomy 16 |
| M30 | SNP array | +1q, -partial 3q, +partial 8q, -partial 12q, -17p |
| M31 | SNP array | +partial 2q, +partial 7p, +partial 7q (x5) |
| M32 | SNP array | -9p (including heterozygous loss of 9p21), -partial 3 (2x, including 3p21), -part1p, +12p, -12q, trisomy 15, -17p, trisomy 21 |
| M33 | SNP array | -9p (including heterozygous loss of 9p21), -1p, -5q, -17p, +6, trisomy 15 |
| M34 | SNP array | -9p (including heterozygous loss of 9p21), -partial 11q |
| M35 | Not performed | - |
| M36 | Not performed | - |
| M37 | SNP array | Monosomy 9 (including homozygous loss of 9p21), monosomy 10, monosomy X |
| M38 | SNP array | Monosomy 9 (including heterozygous loss of 9p21) |
| M39 | SNP array | -1q, -6q, +6p |
| M40 | SNP array | Monosomy 9 (including heterozygous loss of 9p21), partial 1p (3x), - partial 12q, - partial 14q, trisomy 15 |
| M41 | Not performed | - |
| M42 | Not performed | - |
| M43 | SNP array | -partial 9p (including homozygous loss of 9p21), -partial 1p, -partial 4p, -partial 5q (2x), -partial 8q, -12q, -partial 14q, -18p,  -partial 19p, -Xq, +1q, +partial 6p |
| M44 | Not performed | - |
| M45 | Not performed | - |
| M46 | Not performed | - |
| M47 | SNP array | +partial6p, partAI 22q |
| M48 | Not performed | - |
| M49 | Not performed | - |
| M50 | SNP array | Monosomy 9, +6p, -partial 6q, monosomy 10, monosomy 12, monosomy 19 |
| M51 | SNP array | Monosomy 9, +5p, +8q, -12q, trisomy 15, -partial 16q (2x), -17p |
| M52 | SNP array | -partial 3p (including -3p21), monosomy 9 (including homozygous loss of 9p21), monosomy 1, monosomy 14, -partial 2q |

**Table S4.** Logistic regression model of predictors of the composite outcome.

| **Predictor** | ***N*** | **Odds ratio** | **95% confidence interval** | ***P* value** |
| --- | --- | --- | --- | --- |
| *MAP2K1* mutation class | | | | |
| Class I | 20 | Ref. | Ref. | Ref. |
| Class II | 56 | 0.16 | 0.03-0.75 | 0.03 |
| Class III | 26 | 0.31 | 0.05-1.54 | 0.16 |
| *TERT-p* mutations | | | | |
| No | 64 | Ref. | Ref. | Ref. |
| Yes | 38 | 23.1 | 3.99-439.8 | <0.005 |

**Figure S1. Presence of spitzoid histomorphology and fibrosis.**

*
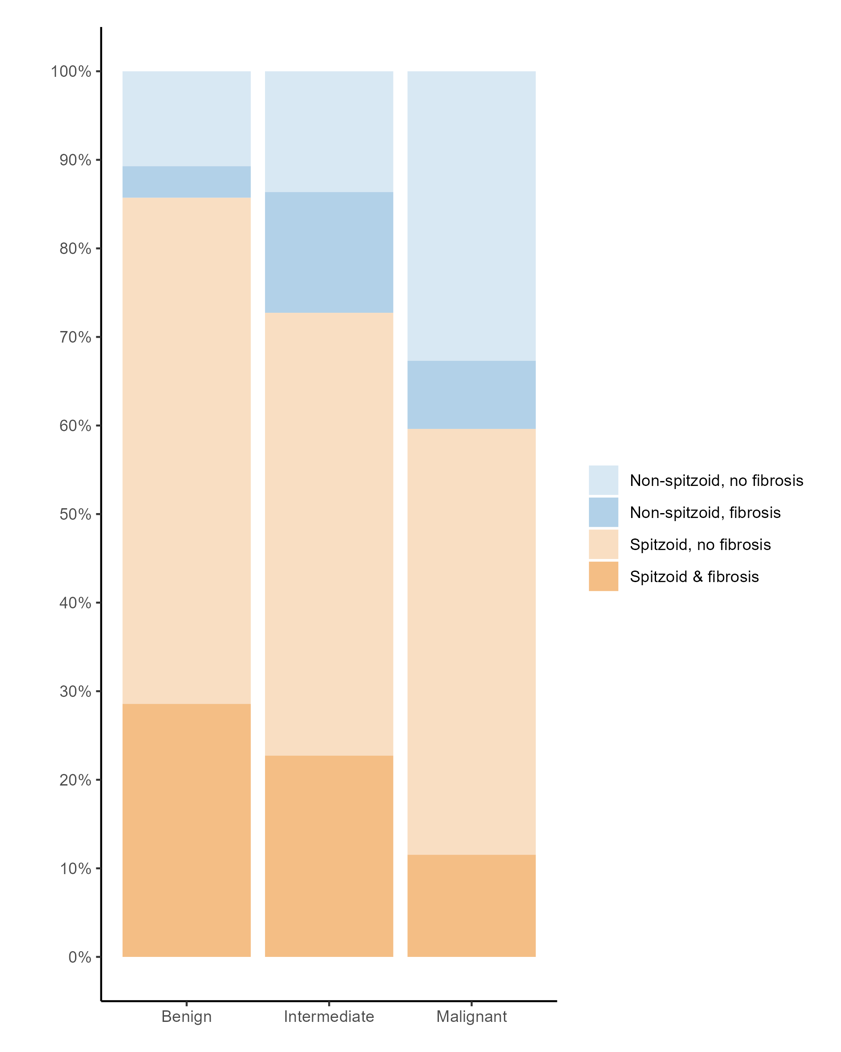

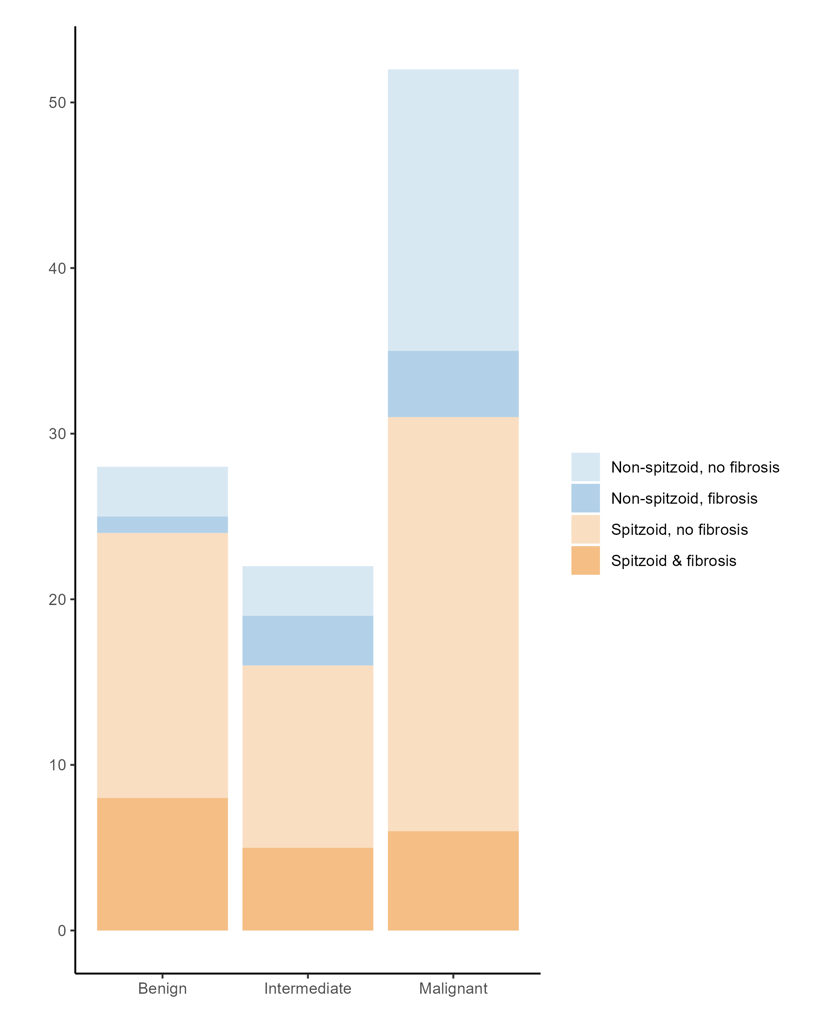
S1A. Absolute numbers.* *S1B. Percentages.*


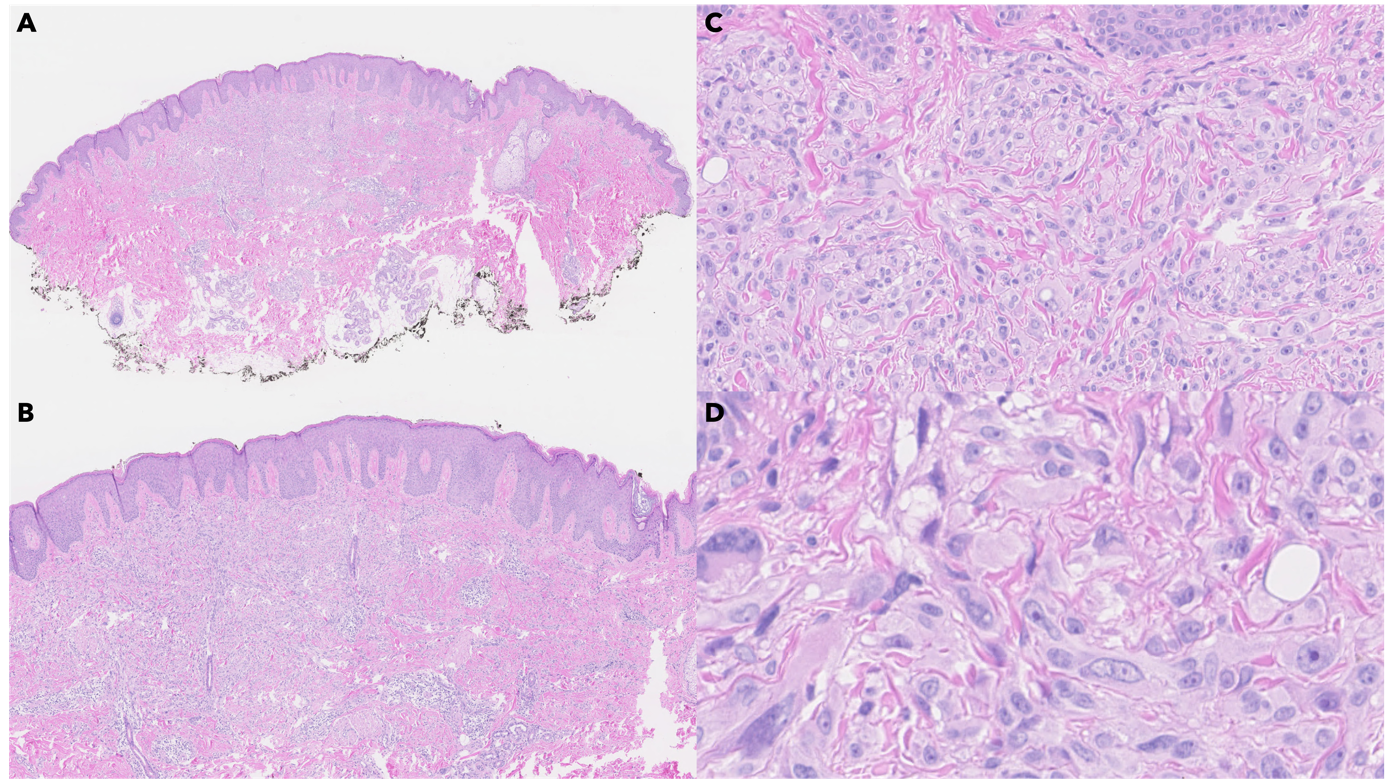
**Figure S2. Histological features of a *MAP2K1*-mutated melanocytoma.**

**A.** Hematoxylin and eosin staining shows a symmetrical, wedge-shaped melanocytic lesion confined to the dermis. **B.** The lesion has moderate cellularity and a desmoplastic stroma, features commonly associated with desmoplastic *HRAS*-mutated Spitz tumors. No significant epidermal involvement or junctional activity is present. **C.** Detail shows nests and individual melanocytes with mild nuclear atypia and presence of nucleoli. The cells display pale eosinophilic cytoplasm. **D.** Detail shows nuclear variation, occasional multinucleation and absence of mitoses. Immunohistochemistry revealed positivity for Melan-A and S100, a low Ki-67 proliferation index, preserved p16 expression, retained BAP1 nuclear expression, and HMB-45 staining with minimal superficial positivity and deeper extinction. Next-generation sequencing (NGS) identified a Class III *MAP2K1^p.I103_K104del^* mutation without additional mutations. SNP array analysis identified two CNVs, including partial loss of chromosomes 2p and 19p, as well as chromothripsis of chromosome 22q. The tumor was classified as *MAP2K1*- mutated melanocytoma.


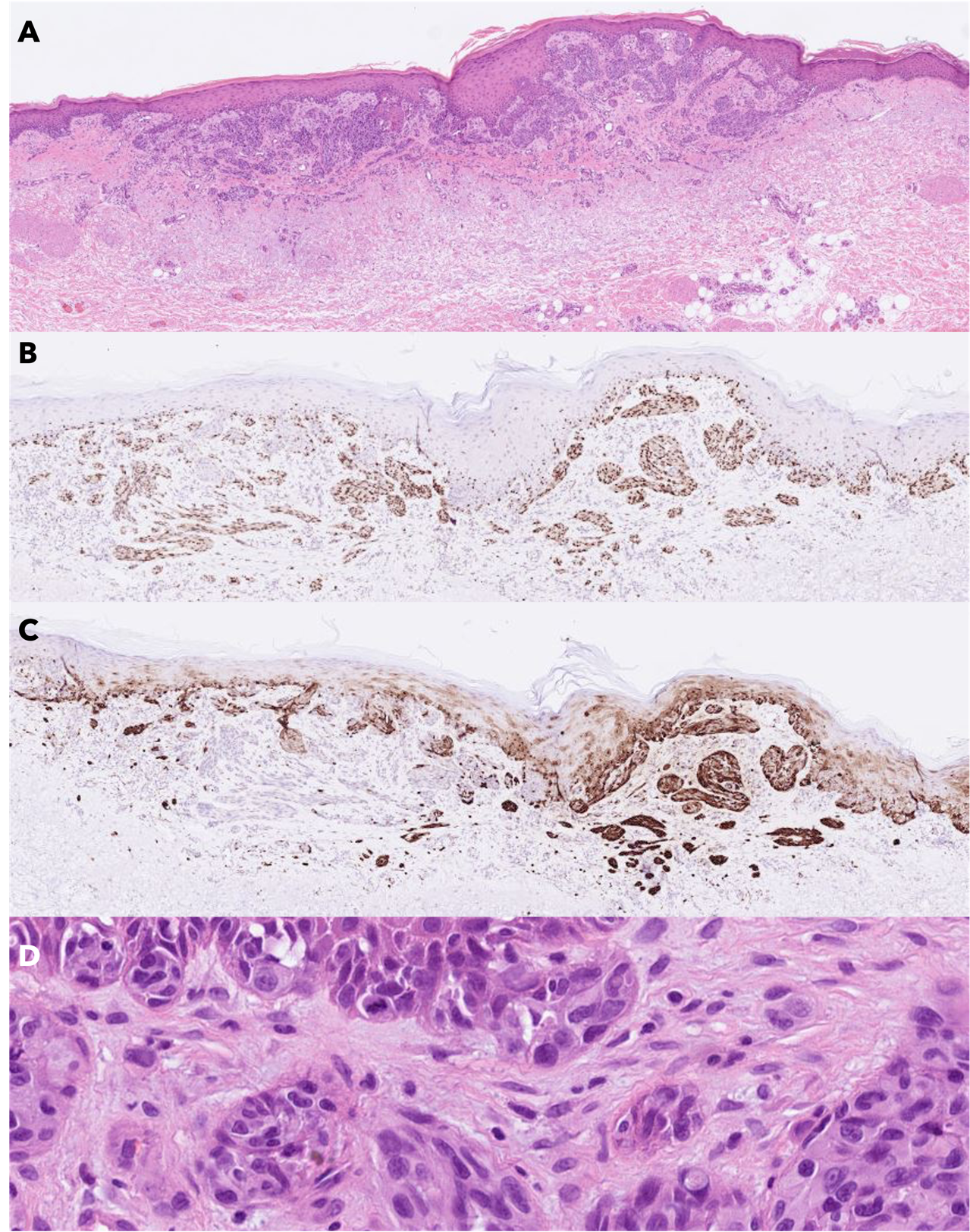
**Figure S3. Histological features of a *MAP2K1*-mutated melanoma.**

**A.** Hematoxylin and eosin staining shows an asymmetrical, compound melanocytic lesion with irregular hyperplasia, areas of epidermal flattening, variable sized junctional nests and irregular dermal nests of melanocytes. **B.** The melanocytes show diffuse and strong PRAME expression **C.** Partial loss of p16 expression (black arrow). **D.** High-power view shows melanocytes with enlarged, vesicular nuclei, central nucleoli, occasional multinucleation, light eosinophilic cytoplasm, and minimal melanin, demonstrating marked cytonuclear atypia and a junctional mitosis. Next generation sequencing identified a *MAP2K1^p.E102_I103delinsVN^* mutation, a *TERT*-promotor mutation (C228T), and a *TP53^E286K^* mutation. Single nucleotide polymorphism array identified 14 segmental copy number variations, including homozygous loss of 9p21 (*CDKN2A*) and partial gain of 6p. The tumor was classified as a superficial spreading melanoma (Breslow thickness 0.7 mm, pT1a).
